# Venom Immunotherapy and Aeroallergen Immunotherapy: How Do Their Outcomes Differ?

**DOI:** 10.3389/falgy.2022.854080

**Published:** 2022-02-16

**Authors:** Cristoforo Incorvaia, Erminia Ridolo, Marina Mauro, Francesco Pucciarini, Enrico Heffler, Giorgio Walter Canonica

**Affiliations:** ^1^Allergy and Clinical Immunology, Medicine and Surgery Department, University of Parma, Parma, Italy; ^2^Allergy Unit, S. Anna Hospital, Como, Italy; ^3^Department of Biomedical Sciences, Humanitas University, Pieve Emanuele, Italy; ^4^Personalized Medicine, Asthma and Allergy, IRCCS Humanitas Research Hospital, Rozzano, Italy

**Keywords:** venom immunotherapy, Hymenoptera, anaphylaxis, prevention, allergy

## Abstract

Allergen immunotherapy (AIT) and venom immunotherapy (VIT) are meant to work on the causes of allergies, respectively, to respiratory allergens and Hymenoptera venom, inducing tolerance to the allergens and modifying the natural history of allergy. Both types of immunotherapies have evidence of efficacy, but actually they present wide differences in both effectiveness and safety. Indeed, as far as the effectiveness of VIT is concerned, if the protection against fatal reactions to stings is considered as the primary objective, more than 40 years of clinical practice demonstrate complete success. The clinical success of AIT is measurable on the basis of reduction or disappearance of allergic symptoms. The difference between the two treatments is even higher as regards safety: AIT has been concerned in the past by a series of fatal reactions caused, which underwent a progressive decrease when it was understood that they were related to the presence of uncontrolled asthma. However, fatal reactions related to failure to recognize the presence of risk factors or administration errors are still reported. Similarly to what has been observed for efficacy, VIT has never been affected by fatal reactions to the administration of venom, and the most important risk of anaphylaxis, which is the concomitance of mastocytosis, is now identified by measuring its marker serum tryptase. To date, mechanisms of hypersensitivity reactions that differentiate respiratory allergy from Hymenoptera venom allergy have not been successfully demonstrated. We have examined the past and present literature in order to propose reasonable hypotheses about the mechanisms actually involved.

## Introduction

Immunotherapy of allergic diseases is currently applied on respiratory allergy, insect venom allergy and food allergy with the aim to induce immunologic tolerance to the causative agent. While immunotherapy for food allergy was only recently approved by FDA, limited to the treatment of peanut allergy (1), those for respiratory allergens and Hymenoptera venom allergy were introduced many years ago, particularly in 1915 (2) and 1930 (3), respectively, when scientific knowledge on pathophysiology of allergy was unknown. Despite its empiric nature, some positive response to the injective administration of grass pollen extracts was observed, paving the way for the progressive development of scientific facts and treatment efficacy. Immunotherapy for insect venom allergy debuted in 1930 (4), but due to the unique natural history of this kind of allergy, which foresees that an initial reaction can be followed by tolerance to subsequent stings of the stinging culprit insect (2), it was believed for 40 years that whole body insect therapy was effective (3). In 1978, a controlled trial comparing whole body treatment to placebo and to insect venom demonstrated the overlap of whole body and placebo, while Hymenoptera venom was extremely effective (4). It is of interest that instead the whole body of fire ant is provably effective (5), but the high disproportion of the ratio between body size and quantity of venom in Hymenoptera and in fire ant can justify this. Immunotherapy with aeroallergens and whit Hymenoptera venom share the effect of not only acting on symptoms, as drugs do, but also on changing the natural history of allergy by modifying its mechanisms and inducing tolerance to the causative allergens (6). Indeed, although some differences between the two treatments are striking, as far as we know there is no literature that has analyzed and compared them. We have dedicated this argument to a comparison of all the divergent aspects between immunotherapy for aeroallergens and immunotherapy for Hymenoptera venom.

## Comparing the Effectiveness

Both types of immunotherapy are acknowledged as effective and disease modifying by the respective consensus documents and guidelines (7, 8). However, their type and degree of effectiveness are not the same (Table 1). Actually, considering the primary objectives, venom immunotherapy (VIT) is aimed at preventing fatal or life-threatening reactions to stings, while allergen immunotherapy (AIT) aims to reduce or abolish allergy symptoms by inducing tolerance. The ability of VIT to prevent fatal reactions to stings must be considered complete, since none has ever been reported in more than 40 years of practice. Furthermore, it has been shown that in patients not fully protected from stings by the generally recommended 100 mcg it is always possible to identify in individual patients a higher dose achieving protection (9). As far as the risk of severe reactions after VIT stopping is concerned, the critical factor is represented by the concomitance of mastocytosis, first reported in 1997, when two patients with mastocysosis had fatal reactions to re-stings from yellow jacket after VIT was stopped based on reaching the recommended duration (10). Now we know that systemic mastocytosis and senior age are major risk factors for severe reactions to stings, which strengthen the indication for VIT. High serum tryptase elevation and mast cell clonality are the most important indicators, but also absence of urticaria/angioedema during sting-induced anaphylaxis may predict a severe reaction (11). As for the duration, it is recommended that it be unlimited (8).

**Table 1:** Features of AIT and VIT.

**Aeroallergen immunotherapy**	**Venom immunotherapy**
**Indication and aim of the treatment**	
In patients with allergic rhinitis and asthma, with the aim to reduce or abolish allergic symptoms.	In patients with hymenoptera venom allergy, with the aim to prevent fatal or life-threatening reactions to stings.
**Route of administration**	
SLIT: drops or tablets SCIT: subcutaneous injections	SCIT: subcutaneous injections
**Dosage**	
Different dosage depending on the allergen and the administration route.	Standardized induction phase (rush or ultra-rush), then maintenance phase 100 mcg/ml every month for the first 3 years and then once every 2 months.
**Treatment duration**	
3–5 years	5 years Lifelong therapy in selected patients (history of severe anaphylaxis, clonal mast cell disorders.
**Safety**	
Reported cases of fatal reactions in the past years, mainly related to SCIT during the induction phase with rush scheme, in patients with uncontrolled asthma, history of previous systemic reactions.	Cases of systemic reactions during the induction phase. No reports of fatal reactions.
**Efficacy**	
Moderate efficacy rate (60–80%)	High efficacy (>90%) Patients still reacting after sting challenges can benefit from increased venom doses.
**Predictors of efficacy**	
*Disease history:* higher efficacy in short term history of AR. *Type of allergen:* higher efficacy in grass pollen and dust mite allergy, lower in allergy to Alternaria, cat or mugwort. Polysensitization and atopic dermatitis are related to lower efficacy. Evaluation of efficacy after the first months (4–6 months) of therapy is a good predictor of overall efficacy after 24–36 months of AIT.	sCD30/TNFRSF8 sTNF-R1

Unlike VIT, which is virtually always effective, the effectiveness of AIT, as well as that of symptomatic drugs, is not predictable in individual patients. In a retrospective study on 1,624 patients suffering from allergic rhinitis (AR) who were treated with AIT and 1,519 matched patients were treated with only symptomatic therapy, symptoms, medications scores and quality of life related to allergic condition before and after treatment were assessed and investigated by cluster analysis. The results showed that AIT was significantly more effective than symptomatic therapy in the treatment of AR, particularly in presence of an association between a better response to AIT and a short-term history of AR with concomitant grass pollen allergy and/or dust mite allergy. Differently, patients with coexisting atopic dermatitis, polysensitization, allergies to cats, Alternaria or mugwort and protracted duration of allergic disease apparently had an unsatisfactory response to AIT (12). Recent studies have added knowledge to the issue, focusing on the most common cause of allergy, namely house dust mites. A prospective study was dedicated to methods for predicting the efficacy of subcutaneous immunotherapy, being observed that the overall efficacy rate at the end of the second year of treatment was 67.4%, and that efficacy of AIT at months 4, 6, 12, and 18 was powerfully associated with efficacy at month 24. In particular, early efficacy (month 4) predicted efficacy at the second year, suggesting the likelihood to determine the need for long-term treatment (13). The other prospective study was aimed at investigating in 154 patients with dust mite-induced AR who had low response to sublingual immunotherapy (SLIT. According to results, 6 months might be a critical time point for assessment of efficacy and dosage adjustment in patients undergoing SLIT, that because of a much higher safety than the subcutaneous route allows a dosage increase in patients with low response, which can may enhance the effectiveness of treatment (14). Another important aspect concerns the qualitative differences between the products of different manufactures. Actually, the need for new products to adhere to requirements from regulatory agencies concerning the quality control (including measurement of protein content, total allergenic activity, and major allergen content) as well as manufacturing is resulting in a major effect on the quality of products (15).

## Comparing the Safety

Likewise for effectiveness, the advantage of VIT over AIT is apparent. In fact, no fatal reactions to VIT have ever been reported (16), although during the buildup phase systemic reactions may occur that hinder the achievement of the maintenance dose, which is recommended in 100 mcg in common patients and 200 mcg in patients exposed to frequent stings, such as beekeepers, or with mastocytosis. This obstacle can be overcome by pharmacological prevention with antihistamines (17) or, in the case of more severe reactions, with omalizumab (18).

Instead, AIT fatalities have been a very serious problem in the past, which today has been significantly reduced but not completely abolished. The origin of the problem dates back to the 1980s, when the introduction of allergen extracts with high biological potency was associated with a series of fatal reactions that imposed limitations, and in some countries abandonment, of AIT (19, 20). The essential understanding that the dominant cause of mortality was injecting the allergen extract to patients with uncontrolled asthma has resulted in a dramatical reduction of the number of fatal reactions, although a slight increase has been observed in recent years (21) suggesting the need for physicians and healthcare professionals to maintain a high level of attention.

## Can Reasonable Hypothesis be Proposed to Explain the Unsettled Differences Between VIT and AIT?

The lesson learned from the large reduction in fatal reactions to AIT achieved by ensuring that at the time of the allergen injection the patient did not have uncontrolled asthma offers us an interpretative key to understand the mechanisms underlying severe reactions to treatment. The mechanisms of action of AIT include the induction of very early desensitization of mast cells and basophils, generation of regulatory T and B regulatory cell responses, regulation of IgE and IgG4, reduction of eosinophils and mast cells in mucosal allergic tissues, and decreases in the activity of basophils in circulation. The key event in inducing tolerance to the administered allergen is the skewing of allergen-specific effector T and effector B cells to a regulatory phenotype and normal immune response to allergens (22). Regarding VIT, it has been found that during ultra-rush schedule T helper type 2 (Th2)-to-Th1 switch occurs, in parallel with natural and acquired regulatory T cell increase. These events occur earlier and at a higher level in less severe subjects, suggesting that VIT tolerance induction is easier to achieve in these patients (23). However, it is imperative to consider the different exposure to allergens in the two sensitization models. In fact, in allergy to inhalant agents, prolonged or even perennial exposure (as occurs with indoor allergens) is mirrored in persistent inflammation, while sporadic exposure related to Hymenoptera stings can result in initial inflammation that relapses with the absence of further stings. In 1994 a study evaluated the importance of the interval between two consecutive stings in influencing the development of venom allergy. The results from 120 allergic patients who experienced a first-time systemic reaction to a sting and 100 controls showed a significant difference in sting-interval distribution indicating that in 60% of allergic patients the sting causing the systemic reaction had been preceded by another, completely tolerated sting not more than 2 months before (24). An assessment of the degree of inflammation was not performed, but it is reasonable to assume that it did not persist beyond the 2 month limit. In contrast, the effects of venom change significantly in subjects exposed to very frequent stings, such as beekeepers. Actually, though the absence of fatal reactions remains, systemic reactions are much more common to bee stings than to vespid stings, as found in a systematic review that reported an incidence of 25.1% for honeybee venom vs. 5.8% for vespid venom (*p* < 0.0001) (25).

A recent study including 21 patients allergic to wasp and/or honey bee venom and 42 healthy participants was aimed to discover new biomarkers of Hymenoptera venom allergy in a group of inflammation factors using multi-marker Bioplex panel and adding the adoption of a novel methodology based on Luminex/xMAP allowed the concurrent determination of serum levels of 37 different inflammatory types. By univariate multivariate statistics, soluble CD30/tumor necrosis factor receptor superfamily, member 8 (sCD30/TNFRSF8), and the soluble tumor necrosis factor receptor 1 (sTNF-R1) cold be considered as effective prognostic factors, their circulating levels being significantly decreased in allergic patients According to the authors, the results shed new light on the allergic inflammatory response to Hymenoptera venom and may contribute to modification and improvement of the diagnostic and monitoring methods (26). However, studies on larger patients' population are needed to confirm the possible usefulness in clinical practice. Finally, a new approach to prevent life-threatening reactions, which is particularly useful for patients who are candidates for AIT but with a history of asthma exacerbations, can be represented by the identification of the responsible mechanisms and their blocking by means of specific biologics, which are the therapeutic innovation of greatest interest (27). Once the cytokine profile has been identified in the individual patient, the specific biologic could be used, as done with omalizumab for patients with repeated systemic reactions to VIT (18) in the initial phase of AIT, to be then suspended when tolerance to treatment is apparent.

## Author Contributions

CI, ER, MM, and GC contributed in ideating the review concept, writing the article, and critically revising the final manuscript. FP and EH contributed in writing the article and critically revising the final manuscript. All authors have read and approved the final version of the manuscript.

## Conflict of Interest

The authors declare that the research was conducted in the absence of any commercial or financial relationships that could be construed as a potential conflict of interest.

## Publisher's Note

All claims expressed in this article are solely those of the authors and do not necessarily represent those of their affiliated organizations, or those of the publisher, the editors and the reviewers. Any product that may be evaluated in this article, or claim that may be made by its manufacturer, is not guaranteed or endorsed by the publisher.
